# Lentinan-functionalized selenium nanoparticles induce apoptosis and cell cycle arrest in human colon carcinoma HCT-116 cells

**DOI:** 10.3389/fnut.2022.987807

**Published:** 2022-08-23

**Authors:** Xiong Gao, Yanting Yao, Xujie Chen, Xiaorong Lin, Xiaobing Yang, Chi-Tang Ho, Bin Li, Zhongzheng Chen

**Affiliations:** ^1^State Key Laboratory of Applied Microbiology Southern China, Guangdong Provincial Key Laboratory of Microbial Safety and Health, Key Laboratory of Agricultural Microbiomics and Precision Application, Ministry of Agriculture and Rural Affairs, Institute of Microbiology, Guangdong Academy of Sciences, Guangzhou, China; ^2^College of Food Science, South China Agricultural University, Guangzhou, China; ^3^Guangdong Yuewei Edible Fungi Technology Co., Ltd., Guangzhou, China; ^4^Department of Food Science, Rutgers University, New Brunswick, NJ, United States; ^5^Guangdong Provincial Key Laboratory of Nutraceuticals and Functional Foods, South China Agricultural University, Guangzhou, China

**Keywords:** lentinan, selenium nanoparticles, characterization, stability, HCT-116, apoptosis, cell cycle arrest

## Abstract

Selenium nanoparticles (SeNPs) have gained extensive attention for their excellent biological activity and low toxicity. However, SeNPs are extremely liable to aggregate into non-bioactive or gray elemental selenium, which limits their application in the biomedicine field. This study aimed to prepare stable SeNPs by using lentinan (LNT) as a template and evaluate its anti-colon cancer activity. The average particle diameter of obtained lentinan-selenium nanoparticles (LNT-SeNPs) was approximately 59 nm and presented zero-valent, amorphous, and spherical structures. The monodisperse SeNPs were stabilized by LNT through hydrogen bonding interactions. LNT-SeNPs solution remained highly stable at 4°C for at least 8 weeks. The stability of LNT-SeNPs solution sharply decreased under high temperature and strong acidic conditions. LNT-SeNPs showed no obvious cytotoxic effect on normal cells (IEC-6) but significantly inhibited the proliferation of five colon cancer cells (HCT-116, HT-29, Caco-2, SW620, and CT26). Among them, LNT-SeNPs exhibited the highest sensitivity toward HCT-116 cells with an IC_50_ value of 7.65 μM. Also, LNT-SeNPs displayed better cancer cell selectivity than sodium selenite and selenomethionine. Moreover, LNT-SeNPs promoted apoptosis of HCT-116 cells through activating mitochondria-mediated apoptotic pathway. Meanwhile, LNT-SeNPs induced cell cycle arrest at G0/G1 phase in HCT-116 cells *via* modulation of cell cycle regulatory proteins. The results of this study indicated that LNT-SeNPs possessed strong potential application in the treatment of colorectal cancer (CRC).

## Introduction

Colorectal cancer (CRC) is one of the most serious and formidable malignancies, with more than 1.88 million (excluding anus) new cases and 915,000 deaths estimated to occur in 2020. Overall, CRC is the third most frequently occurring cancer and the second leading cause of cancer death worldwide (1). In 2020, the incidence and mortality rates of CRC in China increased to 28.8 and 30.6%, respectively (2). Except for genetic factors, about 50% of CRC are estimated to be influenced by dietary factors (3). In recent decades, dietary chemopreventive agents have drawn much attention and may be an effective approach for impeding or delaying the development of CRC (4).

Selenium (Se), an essential trace element, plays an important role in balancing redox systems and regulating immune function (5). Se as a potential chemopreventive agent is gaining considerable attention. Se compounds have been reported to reduce the risk of several cancer types including colorectal, lung, breast, bladder, and prostate cancers (6). Nevertheless, Se displays a very narrow margin between beneficial dose and toxic dose. A high level of Se intake for susceptible patients can result in some symptoms of selenosis (7). Various studies have demonstrated that the chemical forms of Se compounds are closely related to their beneficial and toxic effects (8, 9). Compared with inorganic and organic Se compounds, Selenium nanoparticles (SeNPs) present in the form of zero-valent status exhibit better bioavailability, lower toxicity, and stronger bioactivity (10, 11). However, SeNPs synthesized by redox reactions are usually unstable and liable to aggregate, resulting in a great reduction of bioactivity and bioavailability (5, 12). Therefore, massive efforts have been taken to stabilize SeNPs through the introduction of templates, which can interact with SeNPs and suppress their aggregation. Besides, using bioactive templates will be helpful for regulating or enhancing the bioactivities of SeNPs (13).

Polysaccharides have a wide range of biological activities such as anti-tumor, antioxidation, and immunoregulation (14). Numerous studies have confirmed that various polysaccharides can be used as bioactive templates to stabilize SeNPs. Recent studies showed that SeNPs stabilized by polysaccharides from *Pleurotus tuber-regium*, *Ganoderma lucidum* (15), *Polyporus umbellatus* (10), and *Oudemansiella raphanipies* (16) possessed anti-tumor efficacy. Lentinan (LNT), a natural β-1,3-glucan polysaccharide isolated from *Lentinus edodes*, has been used for adjuvant tumor therapy in Japan and China (17). During 2004–2016, over 17.4% of LNT treatments were given to patients with CRC in China (18). LNT has been regarded as a potential adjuvant for CRC therapy because LNT combined with chemotherapeutics can improve survival rates, enhance immune function, and reduce side effects (19). Jia et al. (20) reported that SeNPs decorated with single-chain LNT (s-LNT) inhibited the proliferation of Hela cells. Liu et al. (21) showed that s-LNT-functionalized SeNPs could effectively inhibit malignant ascites *via* the toll-like receptor 4/TNF receptor-associated factor 3/mitofusin 1 pathway.

However, to our knowledge, the anti-colon cancer activity of lentinan-selenium nanoparticles (LNT-SeNPs) remains virtually unknown. Also, the physicochemical characterization and stability of LNT-SeNPs have not been well-documented. In this study, we synthesized LNT-SeNPs in the redox system and examined its size, morphology, crystal form, elemental composition, valence state, and binding mechanism. Moreover, the stability test was carried out under different temperature, time, and pH conditions. In addition, five colon cancer cell lines (HCT-116, HT-29, Caco-2, SW620, and CT26) combined with a normal small intestine epithelium cell line (IEC-6) were used to evaluate the cytotoxic effects of LNT-SeNPs. Finally, the apoptosis induction and cell cycle arrest of LNT-SeNPs in HCT-116 cells and its underlying molecular mechanisms were further investigated.

## Materials and methods

### Materials and chemicals

LNT and selenomethionine (SeMet) were obtained from Shanghai Yuanye Bio-Technology Co., Ltd. (Shanghai, China). Sodium selenite (Na_2_SeO_3_) and methyl thiazolyl tetrazolium (MTT) were purchased from Sigma-Aldrich (St. Louis, United States). Fetal bovine serum (FBS), Dulbecco’s phosphate-buffered saline (DPBS), Dulbecco’s modified Eagle’s medium (DMEM), RPMI 1640 medium, RIPA buffer, Pierce BCA protein assay kit, eBioscience™ Annexin V apoptosis detection kit, and NuPAGE Bis-Tris gels were obtained from Thermo Scientific (Rockford, United States). Mitochondrial membrane potential (MMP) assay kit and cell cycle analysis kit were obtained from Beyotime Institute of Biotechnology (Shanghai, China). Caspase-9 and –3 colorimetric assay kits were purchased from Nanjing Jiancheng Bioengineering Institute (Nanjing, China). The cytochrome c, Bcl-2, Bax, β-actin, p21, p27, CDK2, and CDK4 antibodies were obtained from Cell Signaling Technology (Boston, United States). The cyclin D1 and CDK6 antibodies were obtained from Abcam (Cambridge, United States).

### Preparation of lentinan-selenium nanoparticles

LNT-SeNPs were prepared according to a previous method (10). Briefly, LNT solution (0–8 mL, 2.5 mg/mL) were mixed with Na_2_SeO_3_ solution (1 mL, 50 mM) under magnetic stirring for 5 min. Then ascorbic acid solution (10 mL, 20 mM) was dropwise added into the mixture. After reconstituting to 20 mL with ultrapure water, the system was stirred for 4 h at 40°C in the dark. Finally, excessive ascorbic acid and Na_2_SeO_3_ were removed by dialysis for 72 h at 4°C.

### Characterization of lentinan-selenium nanoparticles

The concentration of Se was determined by a NexION 350 inductively coupled plasma-mass spectroscopy (ICP-MS, PerkinElmer, United States). The average particle size was measured by a Zetasizer Nano ZS90 particle analyzer (Malvern, United Kingdom). The transmission electron microscopy (TEM) images were carried out with an H-7650 (Hitachi, Japan). The high-resolution TEM (HRTEM) images and corresponding selected area electron diffraction (SAED) pattern were taken on a Talos F200X (FEI, United States) operated at 200 kV. The crystal form was analyzed on a D8 Advance X-ray diffractometer (Bruker, Germany) operated at 40 kV and 40 mA. The patterns were recorded from 5° to 90° at a speed of 10°/min. The valence state was analyzed by an Axis Ultra DLD X-ray photoelectron spectrometer (XPS, Kratos, United Kingdom) equipped with a monochromatic Al Kα X-ray source. The spectra were calibrated with C 1s (284.6 eV). The ultraviolet and visible (UV–Vis) spectra were measured using a U-2910 spectrophotometer (Hitachi, Japan). The Fourier transform infrared spectroscopy (FT-IR) spectra were carried out on a Vertex 70 spectrometer (Bruker, Germany) at the range of 4,000–400 cm^–1^ using the KBr disk method.

### Stability of lentinan-selenium nanoparticles

The stability experiment was explored under different temperature, time, and pH. For temperature, the average particle diameters of LNT-SeNPs were determined after 3 days of storage at 4, 25, 37, and 60°C, respectively. During the 16 weeks of storage at 4°C, the mean diameters of LNT-SeNPs solution were recorded after the 1st, 2nd, 4th, 6th, 8th, 12th, and 16th weeks. For pH levels, the mean diameters of LNT-SeNPs solution were detected after the pH value was adjusted to 2, 4, 6, 7.2, 8, 10, and 12 by HCl or NaOH. The effects of pH value at simulative gastrointestinal environments on the stability of LNT-SeNPs were further evaluated. Firstly, the pH of LNT-SeNPs solution was adjusted to 2 by HCl to mimic gastric environment. The mean diameter was recorded after 1 h incubation at 37°C. Subsequently, the pH of LNT-SeNPs solution was adjusted from 2 to 7.2 by NaOH to simulate intestinal environment. The mean diameter was measured after 2 h incubation at 37°C.

### Cell culture

Human colon cancer cell lines (HCT-116, HT-29, Caco-2, and SW620), mouse colon cancer cell line (CT26), and rat small intestine epithelium cell line (IEC-6) were purchased from the Chinese Academy of Sciences (Shanghai, China). HT-29 and SW620 cells were cultured in a humidified incubator at 37°C with 5% CO_2_ in DMEM containing 10% FBS, penicillin (100 U/mL), and streptomycin (100 U/mL). The other cells were maintained in RPMI 1640 medium.

### Cell viability assay

Cell viability was monitored by the MTT reduction assay (22). Cells (HCT-116, HT-29, Caco-2, SW620, CT26, and IEC-6) were cultivated at a density of 5 × 10^4^ cells/mL on a 96-well plate for 24 h. Then cells were treated with various concentrations (2, 4, 8, 16, 32, and 64 μM) of LNT-SeNPs for another 48 h. Subsequently, the supernatant was removed carefully and 100 μL of MTT (0.25 mg/mL) solution was added into each well. After 2 h incubation, the dark blue formazan crystals were dissolved with 200 μL of dimethyl sulfoxide under gentle shaking. The absorbance at 550 nm was measured with a VersaMax ELISA microplate reader (Molecular Devices, Sunnyvale, United States). Cell viability was determined relative to the control group cultured with medium only (100%). To compare the cytotoxic effects of LNT-SeNPs, Na_2_SeO_3_, and SeMet on HCT-116 and IEC-6 cells, the cell viability was conducted as mentioned above.

### Apoptosis assay

Cell apoptosis was measured by the Annexin V apoptosis detection kit according to the manufacturer’s protocols. After treatment with LNT-SeNPs (3, 6, and 12 μM) for 48 h, HCT-116 cells were harvested and washed with DPBS. Then, the cells were incubated with Annexin V-fluorescein isothiocyanate and propidium iodide (PI) at room temperature in the dark. Stained cells were measured by a CytoFLEX flow cytometer (Beckman Coulter, Brea, United States). Data analysis was performed by CytExpert 2.0 software (Beckman Coulter).

### Determination of mitochondrial membrane potential

The MMP was detected using JC-1 fluorescence probe. After treatment with LNT-SeNPs (3, 6, and 12 μM) for 48 h, HCT-116 cells were collected and incubated with JC-1 based on the manufacturer’s protocols. Stained cells were measured using Beckman flow cytometer. Data analysis was performed by CytExpert 2.0 software (Beckman Coulter).

### Measurement of caspase activity

HCT-116 cells were treated with various concentrations (3, 6, and 12 μM) of LNT-SeNPs for 48 h, and then lysed to obtain the cell lysate. The caspase-9 and –3 activities were measured based on the manufacturer’s instructions.

### Cell cycle assay

The distribution of cell cycle was detected using the cell cycle analysis kit. After treatment with LNT-SeNPs (3, 6, and 12 μM) for 48 h, HCT-116 cells were collected and fixed with precooled 70% ethanol overnight at 4°C. Prior to analysis, cells were washed with DPBS and incubated with PI and RNase A for 0.5 h at room temperature. Stained cells were measured by Beckman flow cytometer. The cell cycle analysis was performed by Flowjo 7.6 software (Tree Star Inc.).

### Western blot analysis

HCT-116 cells were incubated with 3, 6, and 12 μM of LNT-SeNPs for 48 h. Then cells were lysed by RIPA buffer supplemented with protease inhibitors. Protein concentrations were measured by the BCA method. Subsequently, equal amounts of protein were separated by NuPAGE Bis-Tris gels and transferred onto PVDF membranes. After blocking with 5% non-fat milk, the membranes were incubated at 4°C overnight with specific primary antibodies (Bcl-2, Bax, cytochrome c, p21, p27, cyclin D1, CDK2, CDK4, CDK6, and β-actin. Then the membranes were washed three times with TBST and incubated with horseradish peroxidase-conjugated secondary antibodies. Finally, the protein bands were detected with enhanced chemiluminescence reagent using an Omega Lum G imaging system. The relative density of protein bands was normalized to β-actin.

### Statistical analysis

Experimental results were presented as means ± standard deviation (*SD*). Significant differences were analyzed by ANOVA and Duncan’s multiple-range test using SAS 9.2 software (Cary, United States). Data graphs were drawn by Origin 9.0 software (Northampton, United States).

## Results and discussion

### Morphology and physicochemical characterization of lentinan-selenium nanoparticles

**FIGURE 1:**
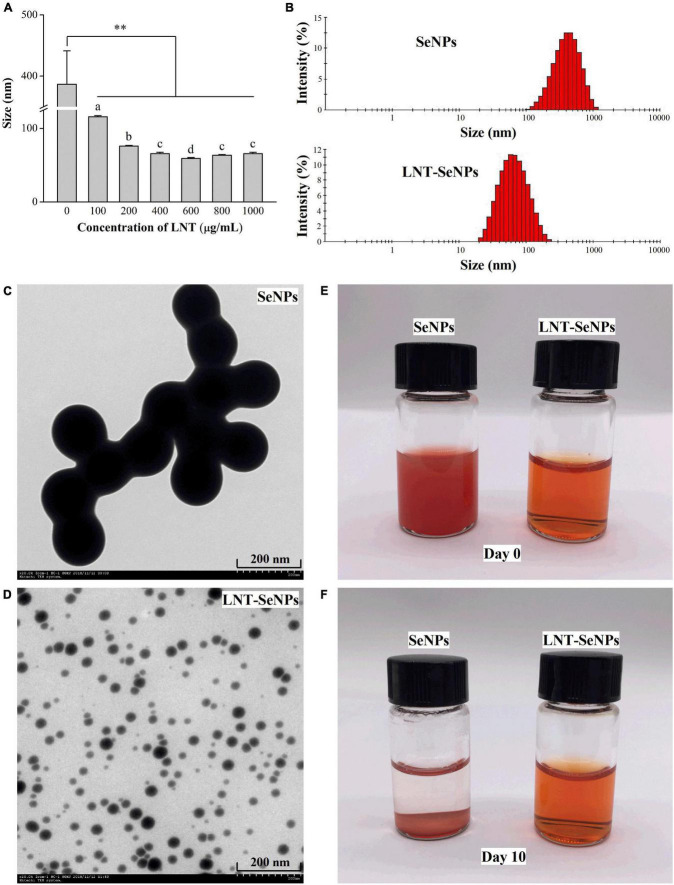
The morphology feature and particle size of SeNPs and LNT-SeNPs. Average particle diameter of SeNPs prepared at different concentrations of LNT **(A)**; size distribution of SeNPs and LNT-SeNPs **(B)**; TEM image of SeNPs **(C)** and LNT-SeNPs **(D)**; photographs of SeNPs and LNT-SeNPs at day 0 **(E)** and day 10 **(F)**. **p < 0.01 represents significant difference between SeNPs alone and SeNPs with LNT as a template. Different letters represent significant difference (*p* < 0.05).

In this study, synthesized SeNPs were decorated by using LNT as a template. As presented in Figure 1A, LNT at the concentration range of 100–1,000 μg/mL significantly decreased the average particle diameter of SeNPs. LNT at 600 μg/mL optimally dropped the mean size from 386.8 to 59.2 nm and the particle size distribution from 105.7–1106.0 to 21.0–220.2 nm (Figure 1B). The Se concentration of LNT-SeNPs solution was about 1.69 mM and the Se content accounted for 18.2%. TEM photographs showed that LNT-SeNPs were monodisperse spherical particles in the solution, while SeNPs with no addition of LNT tended to aggregate and adhere to each other (Figures 1C,D). The particle sizes of LNT-SeNPs and SeNPs observed under the TEM supported the above results of particle analyzer. Additionally, a transparent orange-red solution was obtained in the presence of LNT as a template during the preparation of SeNPs. After storage for 10 days, the LNT-SeNPs solution remained transparent but SeNPs alone appeared obvious precipitation (Figures 1E,F). These findings indicated that the introduction of LNT played a crucial role in the stability of SeNPs.

**FIGURE 2:**
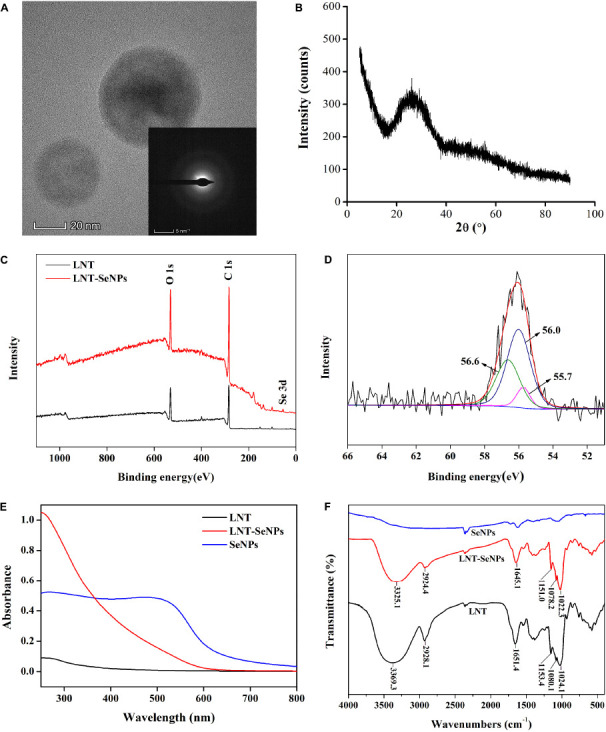
The physicochemical characterization of LNT-SeNPs. HRTEM image of LNT-SeNPs and its corresponding SAED pattern **(A)**; XRD pattern of LNT-SeNPs **(B)**; wide-range XPS patterns of LNT and LNT-SeNPs **(C)**; Se 3d spectra of LNT-SeNPs **(D)**; UV–Vis spectra of LNT, LNT-SeNPs and SeNPs **(E)**; FT-IR spectra of LNT, LNT-SeNPs and SeNPs **(F)**.

The crystal structure of LNT-SeNPs was performed by HRTEM and XRD. As shown in Figure 2A, no lattice fringe and SAED pattern of concentric rings were observed in the HRTEM. Moreover, XRD patterns showed that LNT-SeNPs had broad amorphous bands at lower angles (Figure 2B). These results suggested the amorphous nature of LNT-SeNPs. Consistent with our results, recent studies reported that the amorphous structure of SeNPs stabilized by glucan or chitosan was observed in the XRD patterns (13, 23). The valence state of Se in LNT-SeNPs was analyzed by XPS. An obvious Se 3d peak was found in the XPS spectrum of LNT-SeNPs (Figure 2C). After deconvolution, three Se 3d_5/2_ peaks at 56.6, 56.0, and 55.7 eV were detected (Figure 2D), suggesting the zero-valent Se in LNT-SeNPs. These results confirmed that the amorphous and zero-valent state of LNT-SeNPs were successfully constructed. Several studies pointed out that the amorphous form and zero-valent state of SeNPs were closely related to their excellent bioactivity and low toxicity (23, 24). The UV–Vis and FT-IR spectroscopy were further conducted to confirm the interaction between LNT and SeNPs. As shown in Figure 2E, SeNPs alone exhibited strong absorption bands from 250 and 600 nm while LNT showed weak absorption from 250 to 800 nm. Obviously, the absorption peak of LNT-SeNPs was around 250 nm, which was different from that of LNT and SeNPs. FT-IR spectra of LNT, LNT-SeNPs, and SeNPs were shown in Figure 2F. The adsorption peak of LNT at 3369.3 cm^–1^ was assigned to the –OH stretching vibrations (25), which was shifted to 3325.1 cm^–1^ for LNT-SeNPs, suggesting the strong bonding interaction between hydroxyl groups of LNT and SeNPs. No obvious shifts of other absorption peaks were observed. These results implied that the association of LNT and SeNPs was probably attributed to hydrogen bonding interactions. Similar phenomena were found in previous documents that SeNPs could bind to the hydroxyl groups of polysaccharides from *Polygonatum sibiricum* (26), *Astragalus membranaceus* (27), and black fungus (28).

### The stability of lentinan-selenium nanoparticles affected by temperature, time, and pH

**FIGURE 3:**
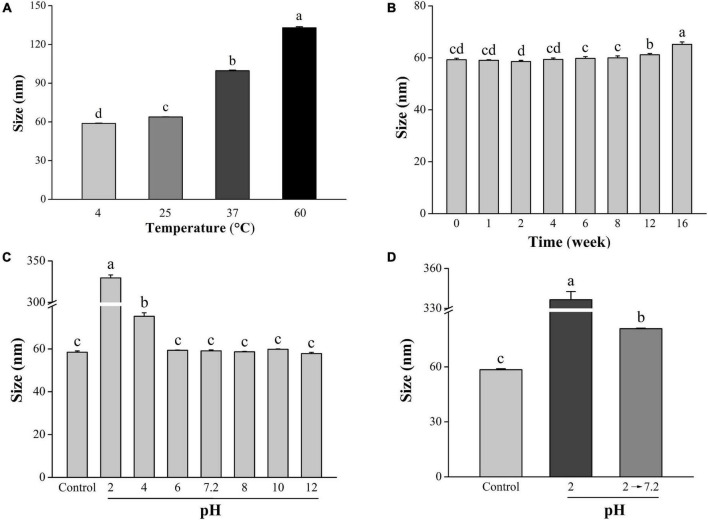
Effects of temperature, storage time, and pH on the stability of LNT-SeNPs. Changes in the average particle diameter of LNT-SeNPs placed in 4, 25, 37, and 60°C for 3 days **(A)**. Changes in the average particle diameter of LNT-SeNPs stored at 4°C for 0–16 weeks **(B)**; effect of pH on the average particle diameter of LNT-SeNPs **(C)**. Effect of pH at simulative gastrointestinal environments on the average particle diameter of LNT-SeNPs **(D)**. Data were shown as mean values ± *SD* (*n* = 3). Different letters represented significant differences (*p* < 0.05).

The stability of LNT-SeNPs is crucial for its extensive application. Herein, we explored the effects of three key factors on the stability of LNT-SeNPs. It could be seen from Figure 3A that the stability of LNT-SeNPs markedly decreased with the rise of storage temperature (4–60°C) after 3 days of storage. Compared with storage at 4°C (58.8 nm), the particle diameter of LNT-SeNPs significantly increased to 63.6, 99.2, and 133.9 nm at 25, 37, and 60°C, respectively. Song et al. (23) reported that SeNPs stabilized by chitosan appeared aggregation and its particle size significantly increased at 70°C, which is consistent with our results. As shown in Figure 3B, the particle size of LNT-SeNPs only slightly increased to 65.2 nm for 16 weeks of storage at 4°C. No obvious change in the particle diameter was observed at the 8th week, which is comparable to that of SeNPs stabilized by *Ulva lactuca* polysaccharide (29). As illustrated in Figure 3C, there was no significant change in the particle diameter of LNT-SeNPs at the pH range of 6–12. Nevertheless, the particle sizes remarkedly increased to 79.2 and 329.5 nm at pH 4 and 2, respectively. Interestingly, the particle size of LNT-SeNPs significantly decreased to 80.8 nm when the pH value was adjusted from 2 to 7.2 (Figure 3D). This phenomenon might be probably attributed to the protonation of LNT under strong acidic condition, which could attenuate the electrostatic interaction between LNT and SeNPs (30).

### Cytotoxic effects of lentinan-selenium nanoparticles on colon cancer cells and normal cells

**FIGURE 4:**
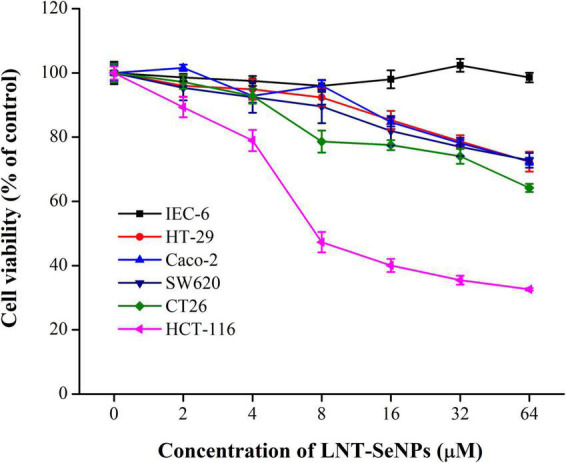
The cytotoxicity of LNT-SeNPs in colon cancer (HCT-116, HT-29, Caco-2, SW620, and CT26) and normal cells (IEC-6) at the concentration range of 2–64 μM. Data were shown as mean values ± *SD* (*n* = 6).

**FIGURE 5:**
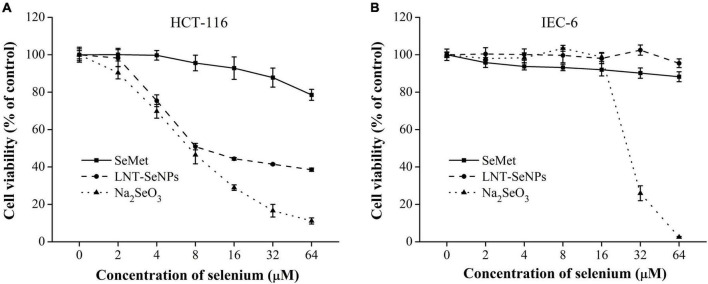
Cytotoxic effects of LNT-SeNPs, Na_2_SeO_3_, and SeMet on HCT-116 **(A)** and IEC-6 cells **(B)** at the concentration range of 2–64 μM. Data were shown as mean values ± *SD* (*n* = 6).

The anti- CRC activity of LNT-SeNPs was screened on HCT-116, HT-29, Caco-2, SW620, CT26, and IEC-6 cells using the MTT method. As shown in Figure 4, LNT-SeNPs displayed obvious anti-proliferative effects on all five cancer cells (HCT-116, HT-29, Caco-2, SW620, and CT26). In contrast, LNT-SeNPs exhibited no significant cytotoxicity toward the normal IEC-6 cells. This specific advantage of LNT-SeNPs might be conducive to reducing its side effects on normal tissues of patients during treatment. A similar result was found by Hu et al. (31) that SeNPs decorated with inulin fructan from *Codonopsis pilosula* had selectivity between cancer and normal cells. Compared with other cancer cells, LNT-SeNPs showed the strongest inhibitory effect on HCT-116 cells with an IC_50_ value of 7.65 μM. Therefore, HCT-116 cells were chosen as a cell model to explore the underlying mechanisms involved in the anti-proliferation of LNT-SeNPs in the subsequent experiment. Moreover, we compared the cytotoxic effect of LNT-SeNPs with Na_2_SeO_3_ and SeMet on HCT-116 and IEC-6 cells. As shown in Figure 5A, LNT-SeNPs and Na_2_SeO_3_ exhibited a better anti-proliferative effect on HCT-116 cells than SeMet. It needed to point out that Na_2_SeO_3_ possessed the strongest anti-proliferation at the Se concentration range of 16–64 μM. However, for normal IEC-6 cells, Na_2_SeO_3_ at 32 and 64 μM also strongly decreased cell viability to 30.0 and 2.5%, respectively. By contrast, the cell viabilities of LNT-SeNPs and SeMet at 64 μM were up to 95.5 and 88.3%, respectively (Figure 5B). These results implied that LNT-SeNPs exhibited better cancer cell selectivity than Na_2_SeO_3_ and SeMet. Consistent findings were also described in previous studies on the anti-proliferation of SeNPs decorated with chitosan (9) and *Polyporus umbellatus* polysaccharide (10).

### Effect of lentinan-selenium nanoparticles on apoptosis in HCT-116 cells

**FIGURE 6:**
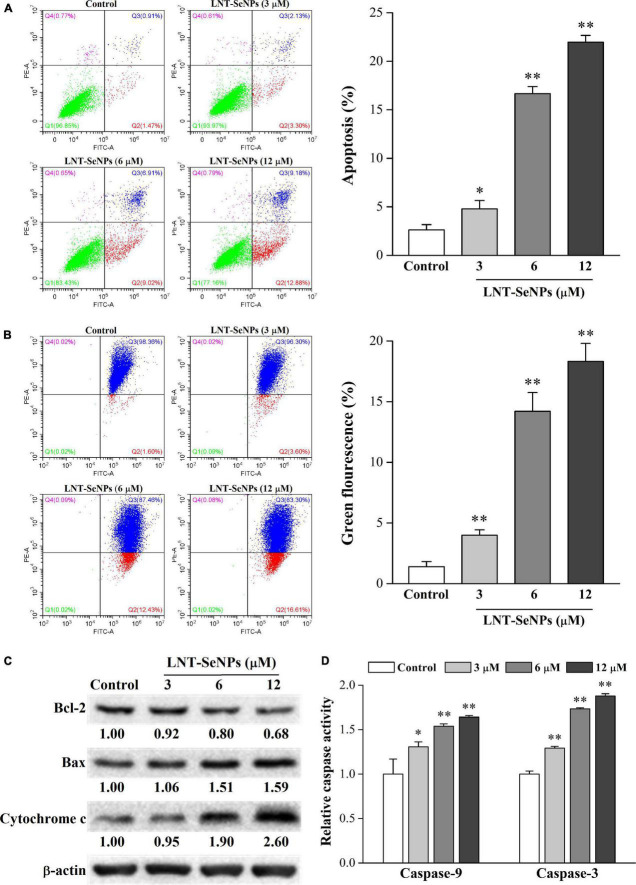
Effects of LNT-SeNPs treatment (3, 6, and 12 μM) on the mitochondria-mediated apoptotic pathway in HCT-116 cells. The apoptosis induction of HCT-116 cells was evaluated by Annexin V/PI double staining **(A)**; the change of MMP was measured by JC-1 staining **(B)**; the protein expression levels of Bcl-2, Bax, and cytochrome c were detected by western blot **(C)**; the caspase-9 and –3 activities were determined by colorimetric assay kit **(D)**. Data were shown as mean values ± *SD* (*n* = 3). *p < 0.05 and **p < 0.01 compared to the control group.

The induction of apoptosis in cancer cells is a promising approach for cancer treatment (32). In this study, the pro-apoptotic effect of LNT-SeNPs on HCT-116 cells was further evaluated by Annexin V/PI double staining method using flow cytometry. As shown in Figure 6A, compared with the control group (2.6%), LNT-SeNPs treatment (3, 6, and 12 μM) significantly increased the total apoptotic percentage of HCT-116 cells to 4.8, 16.7, and 22.0%, respectively. These results indicated that LNT-SeNPs could exert apoptosis induction on HCT-116 cells, which might be partly associated with its anti-proliferation. It has been reported that mitochondria dysfunction can trigger the apoptosis of various cancer cells (33, 34). To explore whether the mitochondria dysfunction was involved in the apoptosis induction of LNT-SeNPs on HCT-116 cells, we determined the change of MMP *via* JC-1 staining assay. As presented in Figure 6B, the green fluorescence in right lower quadrant represented the loss of MMP in HCT-116 cells induced by LNT-SeNPs (34). Compared with the control group, LNT-SeNPs treatment (3, 6, and 12 μM) significantly and dose-dependently induced the loss of MMP in HCT-116 cells, indicating the occurrence of mitochondria dysfunction. The Bcl-2 family members consist of anti-apoptotic (such as Bcl-2) and pro-apoptotic (such as Bax) proteins (35). The up-regulation of Bax/Bcl-2 ratio can increase mitochondrial membrane permeability and the release of cytochrome c. Subsequently, caspase-9 is activated by the released cytochrome c, and then causes the activation of downstream caspase-3, resulting in apoptotic death (36). Our results showed that LNT-SeNPs treatment increased the expression of Bax and cytochrome c and decreased the level of Bcl-2 (Figure 6C). Additionally, LNT-SeNPs significantly induced a dose-dependent increase in the activities of caspase-9 and –3 in comparison to the control group (Figure 6D). These results indicated that LNT-SeNPs could induce apoptosis of HCT-116 cells *via* mitochondria-mediated intrinsic pathway.

### Effect of lentinan-selenium nanoparticles on cell cycle arrest in HCT-116 cells

**FIGURE 7:**
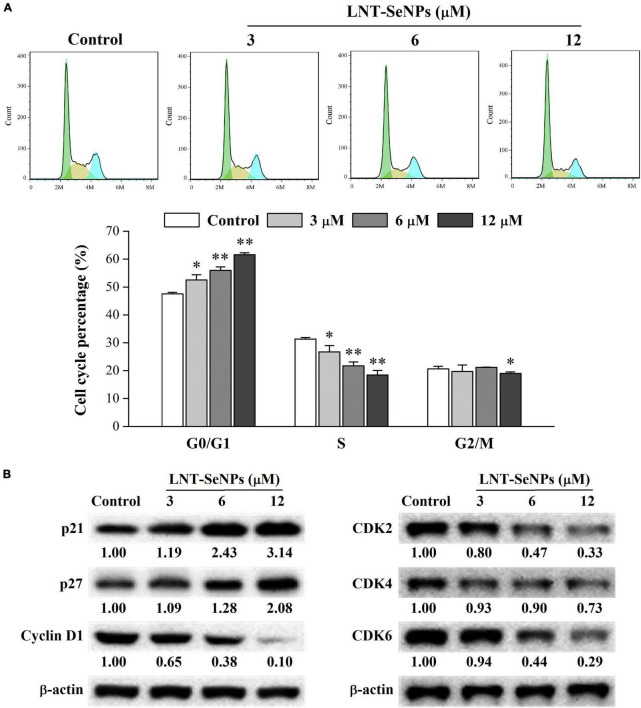
Effects of LNT-SeNPs treatment (3, 6, and 12 μM) on the cell cycle progression in HCT-116 cells. The cell cycle distribution of HCT-116 cells was analyzed by the PI staining method **(A)**; the protein expression levels of p21, p27, cyclin D1, CDK2, CDK4, and CDK6 were detected by western blot **(B)**. Data were shown as mean values ± *SD* (*n* = 3). *p < 0.05 and **p < 0.01 compared to the control group.

Apart from apoptosis, cell cycle arrest is another crucial approach for preventing the proliferation of cancer cells (37). Thus, the cell cycle distribution of HCT-116 cells affected by LNT-SeNPs was further explored using flow cytometry. As presented in Figure 7A, LNT-SeNPs treatment significantly increased cell population in the G0/G1 phase in a dose-dependent manner compared with the control group. LNT-SeNPs at 12 μM induced G0/G1 phase arrest from 47.5 to 61.6% in HCT-116 cells. As a result of G0/G1 phase arrest, the proportions of cells in S and G2/M phases were remarkedly decreased from 31.3 to 18.5% and from 20.6 to 19.0%, respectively. These results suggested that the anti-proliferative activity of LNT-SeNPs in HCT-116 cells might be partly caused by G0/G1 phase cell arrest. However, Huang et al. (38) reported that *Pleurotus tuber-regium*-conjugated SeNPs induced G2/M phase arrest in HCT-116 cells. The inconsistent results might be related to different templates used to stabilize SeNPs. The cell cycle is primarily driven by cyclin-dependent kinases (CDKs) and cyclins (39). The complexes including cyclin D–CDK4/6 and cyclin E–CDK2 drive the transition from G1 to S phase (40). The cyclin D–CDK4/6 is essential for entering G1 phase, while the cyclin E–CDK2 regulates the induction of DNA synthesis in late G1 and early S phases (37). The INK4 proteins specifically interfere with the association between cyclin D and CDK4/6, whereas the Cip/Kip family of inhibitors such as p21 and p27 inhibit cyclin E–CDK2 activity (41). To explore the molecular mechanism of LNT-SeNPs on cell cycle arrest in HCT-116 cells, the protein expression levels of p21, p27, cyclin D1, CDK2, CDK4, and CDK6 were detected by western blot. As shown in Figure 7B, LNT-SeNPs treatment (3, 6, and 12 μM) exhibited a dose-dependent increase in the expression levels of p21 and p27 proteins in HCT-116 cells. Moreover, the down-regulation of cyclin D1, CDK2, CDK4, and CDK6 proteins was observed after LNT-SeNPs treatment. These results confirmed that LNT-SeNPs could arrest HCT-116 cells at G0/G1 phase through modulation of cell cycle regulatory proteins. Several studies have demonstrated that these cell cycle regulatory proteins are closely related to the G0/G1 phase arrest in cancer cells. Xia et al. (42) found that anisomycin-loaded functionalized SeNPs up-regulated the protein levels of p21 and p27 and arrested the cell cycle progression at the G0/G1 phase in HepG2 cells. Lee et al. (43) reported that cannabidiol significantly induced G1 phase arrest in SW620, SW480, and HCT-116 cells by down-regulating the protein levels of cyclin D1, cyclin D3, CDK2, CDK4, and CDK6.

## Conclusion

In the present study, SeNPs stabilized by LNT with a mean size of ∼59 nm and could bind with LNT *via* hydrogen bonding interactions. The well-dispersed LNT-SeNPs presented zero-valent, amorphous, and spherical structures and had good stability at 4°C. Compared with SeMet and Na_2_SeO_3_, LNT-SeNPs exhibited good selectivity between cancer and normal cells. Among five colon cancer cells, LNT-SeNPs showed the highest sensitivity toward HCT-116 cells. Moreover, LNT-SeNPs inhibited the anti-proliferation of HCT-116 cells by regulating mitochondria-mediated apoptotic pathway and inducing cell cycle arrest at G0/G1 phase. The present study provides valuable scientific evidence for the application of LNT-SeNPs in the chemoprevention of CRC.

## Data availability statement

The raw data supporting the conclusions of this article will be made available by the authors, without undue reservation.

## Author contributions

XG: writing – original draft, methodology, software, data curation, and funding acquisition. YY and XC: investigation. XL: methodology and validation. XY: funding acquisition. C-TH: writing – review and editing. BL and ZC: funding acquisition and supervision. All authors contributed to the article and approved the submitted version.
